# Supplementary material for: The representational space of observed actions
Source: eLife. 2019 Dec 5;8:e47686. doi: 10.7554/eLife.47686 (PMC6894926; doi:10.7554/eLife.47686)
Supplement: Supplementary file 1. — Cluster table standard RSA (semantic model). List of clusters resulting from the standard RSA for the semantic model which survived correction for multiple comparisons (cluster p-value<0.05; see Materials and methods and Figure 4). Coordinates are in MNI space. Labels are based on MRI scans that originated from the OASIS project (http://www.oasis-brains.org/) and were provided by Neuromorphometrics, Inc (http://www.neuromorphometrics.com/) under academic subscription provided in SPM12 and Glasser’s surface-based atlas (Glasser et al., 2016). [file elife-47686-supp1.docx]

| **Model** |  | **max T** | **coordinates** | | | **Neuromorphometrics*** | **Glasser** |
| --- | --- | --- | --- | --- | --- | --- | --- |
|  |  |  | *x* | *y* | *z* |  |  |
| semantic | |  |  |  |  |  |  |
|  |  | 9.487 | -44.90 | -73.39 | 16.02 | Left MOG middle occipital gyrus | Area TemporoParietoOccipital Junction 3 |
|  |  | 8.696 | 50.93 | -58.71 | 5.63 | Right MTG middle temporal gyrus | Area TemporoParietoOccipital Junction 2 |
|  |  | 6.941 | -3.40 | -97.73 | 6.23 | Left OCP occipital pole | Primary Visual Cortex |
|  |  | 5.496 | -41.71 | 5.83 | 26.42 | Left PrG precentral gyrus | Area IFJp |
|  |  | 4.849 | -6.29 | -56.28 | 53.93 | Left PCu precuneus | PreCuneus Visual Area |
|  |  | 4.739 | -56.34 | -42.13 | 29.73 | Left SMG supramarginal gyrus | Area PF Complex |
|  |  | 4.615 | 29.31 | -77.04 | 27.38 | Right SOG superior occipital gyrus | Seventh Visual Area |
|  |  | 4.578 | 22.95 | 1.36 | 60.40 | Right SFG superior frontal gyrus | Area 6 anterior |
|  |  | 4.572 | 46.16 | 3.29 | 45.88 | Right PrG precentral gyrus | Area 55b |
|  |  | 4.397 | -26.36 | -5.64 | 49.76 | Left MFG middle frontal gyrus | Area 6 anterior |
|  |  | 4.365 | 34.08 | -45.65 | 48.61 | Right SPL superior parietal lobule | Anterior IntraParietal Area |
|  |  | 4.226 | -29.95 | -56.81 | 46.42 | Left SPL superior parietal lobule | Area Lateral IntraParietal dorsal |

***Supplementary file 1. Cluster table standard RSA (semantic model).*** *List of clusters resulting from the standard RSA for the semantic model which survived correction for multiple comparisons (cluster p-value < 0.05; see Methods and Figure 4). Coordinates are in MNI space. Labels are based on MRI scans that originated from the OASIS project (*[*http://www.oasis-brains.org/)*](http://www.oasis-brains.org/)) *and were provided by Neuromorphometrics, Inc. (http://*[*www.neuromorphometrics.com/)*](http://www.neuromorphometrics.com/)) *under academic subscription provided in SPM12 and the Glasser’s surface-based atlas^18^.*
