# Supplementary material for: The representational space of observed actions
Source: eLife. 2019 Dec 5;8:e47686. doi: 10.7554/eLife.47686 (PMC6894926; doi:10.7554/eLife.47686)
Supplement: Supplementary file 2. — Cluster table multiple regression RSA. List of clusters resulting from the multiple regression RSA for the eight different models (semantic, body, movement, object, transitivity, distance, 1 vs 2 people, HMAX-C1) which survived correction for multiple comparisons (cluster p-value<0.05; see Materials and methods and Figure 6 and Figure 6—figure supplement 1). Coordinates are in MNI space. Labels are based on MRI scans that originated from the OASIS project (http://www.oasis-brains.org/) and were provided by Neuromorphometrics, Inc (http://www.neuromorphometrics.com/) under academic subscription provided in SPM12 and Glasser’s surface-based atlas (Glasser et al., 2016). [file elife-47686-supp2.docx]

| **Model** | **max T** | **coordinates** | | | **Glasser et al., 2016** | **Neuromorphometrics*** |
| --- | --- | --- | --- | --- | --- | --- |
|  |  | x | y | z |  |  |
| semantic | 4.91 | -43.87 | -66.50 | 13.44 | L_MST_ROI | Left AnG angular gyrus |
|  |  |  |  |  |  |  |
| body | 4.56 | 48.55 | -58.77 | 8.99 | R_TPOJ2_ROI | Right MTG middle temporal gyrus |
|  | 5.58 | -52.15 | -54.66 | 23.98 | L_PGi_ROI | Left AnG angular gyrus |
|  |  |  |  |  |  |  |
| movement | 6.02 | -43.52 | -75.13 | 3.94 | L_MT_ROI | Left IOG inferior occipital gyrus |
|  | 6.60 | -25.27 | -63.92 | 45.29 | L_MIP_ROI | Left SPL superior parietal lobule |
|  | 6.89 | 47.78 | -60.69 | 6.50 | R_MST_ROI | Right MTG middle temporal gyrus |
|  |  |  |  |  |  |  |
| object | 4.69 | 25.71 | -68.64 | 42.52 | R_IPS1_ROI | Right SPL superior parietal lobule |
|  | 5.10 | -26.35 | -55.37 | -11.91 | L_VMV3_ROI | Left FuG fusiform gyrus |
|  |  |  |  |  |  |  |
| transitivity | 5.10 | -45.16 | -76.17 | 6.40 | L_MT_ROI | Left IOG inferior occipital gyrus |
|  |  |  |  |  |  |  |
| distance | 5.58 | -18.35 | -98.36 | 7.12 | L_V2_ROI | Left OCP occipital pole |
|  | 5.65 | -43.51 | -71.28 | 9.31 | L_MT_ROI | Left MOG middle occipital gyrus |
|  | 10.05 | 44.72 | -65.94 | 6.93 | R_MST_ROI | Right IOG inferior occipital gyrus |
|  | 4.72 | 13.44 | -53.32 | 64.42 | R_7AR_ROI | Right SPL superior parietal lobule |
|  | 5.44 | -42.72 | -50.71 | -14.51 | L_TE2p_ROI | Left FuG fusiform gyrus |
|  |  |  |  |  |  |  |
| 1vs2People | 4.59 | -14.21 | -101.80 | 5.49 | L_V2_ROI | Left OCP occipital pole |
|  | 4.77 | 3.68 | -84.13 | -3.01 | R_V1_ROI | Right LiG lingual gyrus |
|  | 7.30 | -45.18 | -74.63 | 15.44 | L_LO3_ROI | Left MOG middle occipital gyrus |
|  | 4.81 | 30.33 | -70.91 | 28.78 | R_IPS1_ROI | Right SOG superior occipital gyrus |
|  | 6.90 | 46.32 | -67.94 | 6.51 | R_MT_ROI | Right IOG inferior occipital gyrus |
|  | 5.65 | 28.81 | -67.84 | -12.04 | R_V8_ROI | Right OFuG occipital fusiform gyrus |
|  | 7.11 | 4.96 | -55.94 | 15.10 | R_v23ab_ROI | Right PCu precuneus |
|  | 5.58 | -54.97 | -50.09 | 15.93 | L_STV_ROI | Left STG superior temporal gyrus |
|  |  |  |  |  |  |  |
| HMAX-C1 | 5.36 | -6.12 | -91.32 | -5.02 | L_V1_ROI | Left Calc calcarine cortex |
|  | 6.80 | 22.30 | -82.74 | -11.47 | R_V3_ROI | Right OFuG occipital fusiform gyrus |
|  | 4.63 | 46.84 | -67.60 | 9.09 | R_MT_ROI | Right IOG inferior occipital gyrus |
|  | 6.40 | 22.01 | -43.44 | -9.96 | R_VMV1_ROI | Right LiG lingual gyrus |
|  | 4.65 | -32.53 | -38.72 | -13.11 | L_PHA3_ROI | Left FuG fusiform gyrus |
|  |  |  |  |  |  |  |

***Supplementary file 2. Cluster table multiple regression RSA.*** *List of clusters resulting from the multiple regression RSA for the eight different models (semantic, body, movement, object, transitivity, distance, 1 vs 2 people, HMAX-C1) which survived correction for multiple comparisons (cluster p-value < 0.05; see Methods and Figures 6 and Figure 6 – figure supplement 1). Coordinates are in MNI space. Labels are based on MRI scans that originated from the OASIS project (*[*http://www.oasis-brains.org/)*](http://www.oasis-brains.org/)) *and were provided by Neuromorphometrics, Inc. (http://*[*www.neuromorphometrics.com/)*](http://www.neuromorphometrics.com/)) *under academic subscription provided in SPM12 and the Glasser’s surface-based atlas^18^.*
