# Supplementary material for: The representational space of observed actions
Source: eLife. 2019 Dec 5;8:e47686. doi: 10.7554/eLife.47686 (PMC6894926; doi:10.7554/eLife.47686)
Supplement: Supplementary file 3. — We initially kept actions that were mentioned by at least 20% of the participants. The final selected twenty-eight actions used for the study are the ones highlighted. See Materials and methods section for the procedure used to select these actions. [file elife-47686-supp3.docx]

| **Actions** |  |  |  |
| --- | --- | --- | --- |
| **biking** | grocery shopping | **reading** | **walking** |
| brushing teeth | **handshaking** | **running** | **washing hands** |
| **combing hair** | **holding hands** | singing | **washing the dishes** |
| cleaning the floor | **hugging** | **sleeping** | **watching tv** |
| **cutting** | **listening to music** | **swimming** | watering plants |
| **dancing** | making coffee | switching on light | **waving** |
| **drinking** | **opening door** | **taking a shower** | **writing** |
| **driving** | **painting** | taking the train |  |
| **eating** | **playing instrument** | **talking on the phone** |  |
| getting dressed | **playing videogames** | **talking** |  |

**Supplementary file 3. *List of actions identified using the online survey.*** *We initially kept actions that were mentioned by at least 20% of the participants. The final selected twenty-eight actions used for the study are the ones highlighted. See Methods section for the procedure used to select these actions.*
