# Supplementary material for: The representational space of observed actions
Source: eLife. 2019 Dec 5;8:e47686. doi: 10.7554/eLife.47686 (PMC6894926; doi:10.7554/eLife.47686)
Supplement: Transparent reporting form [file elife-47686-transrepform.pdf]

eLIFE
1st Floor
24 Hills Road
Cambridge CB2 1JP, UK
P 01223 855340
W elifesciences.org
T @elife

eLife's transparent reporting form

We encourage authors to provide detailed information *within their submission* to facilitate the interpretation and replication of experiments. Authors can upload supporting documentation to indicate the use of appropriate reporting guidelines for health-related research (see EQUATOR Network), life science research (see the BioSharing Information Resource), or the ARRIVE guidelines for reporting work involving animal research. Where applicable, authors should refer to any relevant reporting standards documents in this form.

If you have any questions, please consult our Journal Policies and/or contact us: editorial@elifesciences.org.

Sample-size estimation

- You should state whether an appropriate sample size was computed when the study was being designed
- You should state the statistical method of sample size computation and any required assumptions
- If no explicit power analysis was used, you should describe how you decided what sample (replicate) size (number) to use

Please outline where this information can be found within the submission (e.g., sections or figure legends), or explain why this information doesn't apply to your submission:

We did not conduct a formal power analysis for the fMRI experiment. The sample size (N=20 volunteers) was decided based on: 1) previous similar fMRI studies (e.g. Bracci et al., 2016); 2) the need of counterbalancing the presentation order of the tasks in the behavioral studies 3) and within the constraints of the funding available.
We also conducted three behavioral studies (N=37, N=15, and N=15 respectively) before the main experiment to select the stimuli to use. Detailed information can be found in the Methods section (*Participants* and *Stimulus selection* subsections)

Replicates

- You should report how often each experiment was performed
- You should include a definition of biological versus technical replication
- The data obtained should be provided and sufficient information should be provided to indicate the number of independent biological and/or technical replicates
- If you encountered any outliers, you should describe how these were handled
- Criteria for exclusion/inclusion of data should be clearly stated
- High-throughput sequence data should be uploaded before submission, with a private link for reviewers provided (these are available from both GEO and ArrayExpress)

Please outline where this information can be found within the submission (e.g., sections or figure legends), or explain why this information doesn't apply to your submission:

The fMRI experiment was performed just once and was followed by one behavioral session. In order to select actions suitable for the fMRI experiment, prior to the fMRI experiment we conducted one online experiment (N=37) and two behavioral experiments (N=15 in both experiments), using different groups of participants and different stimuli. No outliers were encountered, and therefore all data were used for the analysis. In order to participate, only participants with no history of neurological or psychiatric disease were selected. Further information can be found in the *Methods* section (*Participants* and *Stimulus selection* subsections).

Statistical reporting

- Statistical analysis methods should be described and justified
- Raw data should be presented in figures whenever informative to do so (typically when N per group is less than 10)
- For each experiment, you should identify the statistical tests used, exact values of N, definitions of center, methods of multiple test correction, and dispersion and precision measures (e.g., mean, median, SD, SEM, confidence intervals; and, for the major substantive results, a measure of effect size (e.g., Pearson's r, Cohen's d)
- Report exact p-values wherever possible alongside the summary statistics and 95% confidence intervals. These should be reported for all key questions and not only when the p-value is less than 0.05.

Please outline where this information can be found within the submission (e.g., sections or figure legends), or explain why this information doesn't apply to your submission:

We provide a detailed description of the methods and data analysis conducted in the Methods section. In addition, information about the statistical test used is also provided in the Figure captions of the main text and of the supplementary information.

(For large datasets, or papers with a very large number of statistical tests, you may upload a single table file with tests, Ns, etc., with reference to sections in the manuscript.)

Group allocation

- Indicate how samples were allocated into experimental groups (in the case of clinical studies, please specify allocation to treatment method); if randomization was used, please also state if restricted randomization was applied
- Indicate if masking was used during group allocation, data collection and/or data analysis

Please outline where this information can be found within the submission (e.g., sections or figure legends), or explain why this information doesn't apply to your submission:

Participants were not allocated in different experimental groups. All participants took part in the same experimental conditions.

Additional data files ("source data")

- We encourage you to upload relevant additional data files, such as numerical data that are represented as a graph in a figure, or as a summary table
- Where provided, these should be in the most useful format, and they can be uploaded as "Source data" files linked to a main figure or table
- Include model definition files including the full list of parameters used
- Include code used for data analysis (e.g., R, MatLab)
- Avoid stating that data files are "available upon request"

Please indicate the figures or tables for which source data files have been provided:

Stimuli and source data used to plot Figure 3, 4, 5, 6 and 7 can be found in OSF.io (https://osf.io/cvrb2/?view_only=cb1628c4ad4346119c9d4da46e1b66f8)
